# Novel monkey mAbs induced by a therapeutic vaccine targeting the hepatitis B surface antigen effectively suppress hepatitis B virus in mice

**DOI:** 10.1093/abt/tbab020

**Published:** 2021-09-29

**Authors:** Yuanzhi Chen, Xinchu Xiang, Ruoyao Qi, Yiwen Wang, Yang Huang, Min You, Yangfei Xian, Yangtao Wu, Rao Fu, Ciming Kang, Jixian Tang, Hai Yu, Tianying Zhang, Quan Yuan, Wenxin Luo, Ningshao Xia

**Affiliations:** 1 State Key Laboratory of Molecular Vaccinology and Molecular Diagnostics, School of Public Health and School of Life Science, Xiamen University, Xiamen 361102, China; 2 National Institute of Diagnostics and Vaccine Development in Infectious Diseases, School of Public Health and School of Life Science, Xiamen University, Xiamen 361102, China

**Keywords:** chronic hepatitis B infection, hepatitis B surface antigen, B cell culture, cynomolgus monkey antibody, antibody-mediated HBV suppression

## Abstract

**Background:**

We have previously obtained a mouse anti-hepatitis B surface antigen (HBsAg) antibody E6F6 with long-lasting serum HBsAg clearance effects. The E6F6 epitope-based protein CR-T3-SEQ13 (HBsAg aa 113-135) vaccination therapy in cynomolgus monkeys induced long-term polyclonal antibodies-mediated clearance of HBsAg in the HBV transgenic (HBV-Tg) mice.

**Methods:**

We isolated monoclonal antibodies from CR-T3-SEQ13 vaccinated cynomolgus monkeys, compared their therapeutic effects with E6F6, identified their epitopes on HBsAg, determined the pharmacokinetics and studied their physical property.

**Results:**

A panel of anti-HBsAg mAbs was generated through memory B cell stimulatory culture. Two lead monkey-human chimeric antibodies, C1-23 and C3-23, effectively suppressed HBsAg and HBV DNA in HBV-Tg mice. The humanized antibodies and humanized-mouse reverse chimeric antibodies of two antibodies exhibited comparable HBsAg clearance and viral suppression efficacy as those versions of E6F6 in HBV-Tg mice. Humanized antibody hu1-23 exhibited more efficacy HBsAg-suppressing effects than huE6F6-1 and hu3-23 in HBV-Tg mice at dose levels of 10 and 20 mg/kg. Evaluation of the binding sites indicates that the epitope recognized by hu1-23 is located in HBsAg aa 118-125 and 121-125 for hu3-23. Physical property study revealed that hu1-23 and hu3-23 are stable enough for further development as a drug candidate.

**Conclusions:**

Our data suggest that the CR-T3-SEQ13 protein is a promising HBV therapeutic vaccine candidate, and hu1-23 and hu3-23 are therapeutic candidates for the treatment of chronic hepatitis b. Moreover, the generation of antibodies from the epitope-based vaccinated subjects may be an alternative approach for novel antibody drug discovery.

**Statement of Significance**

Cynomolgus monkey mAbs were generated from an HBsAg-epitope-based protein vaccination through memory B cell stimulatory culture. The humanized antibodies can efficiently mediate HBsAg clearance in HBV-Tg mice and may serve as anti-HBsAg therapeutic candidates. The generation of mAbs from the epitope-based vaccinated subjects is an alternative approach for novel antibody discovery.

## INTRODUCTION

Despite the availability of effective vaccines, hepatitis B virus (HBV) infection remains a major global health problem with high morbidity and mortality. Chronic hepatitis B (CHB) affects the liver and can occur as an acute or chronic disease. It is estimated that about 257–291 million individuals are chronically infected with HBV worldwide, which causes approximately 780 000 deaths from HBV-related diseases per year [1, 2]. The optimal endpoint of treatment for chronic HBV infection is clearance of hepatitis B surface antigen (HBsAg). However, the currently approved anti-HBV drugs rarely achieve this endpoint [2]. Therapeutic antibodies have experienced exponential growth as a new drug modality for treating various human diseases, including cancers, autoimmune, metabolic and infectious diseases [3]. The US Food and Drug Administration has approved several therapeutic antibody drugs to treat various infectious diseases such as inhalational anthrax, respiratory syncytial virus and human immunodeficiency virus (HIV) [4–6]. In recent years, novel mAb-based immunotherapy has represented a promising new modality for treating chronic HBV infection. Several mAbs against HBsAg or PreS1 were reported to protect against HBV infection, neutralize or clear circulating HBV [7–11].

A mouse mAb E6F6, with long-lasting serum HBsAg clearance effects, was generated by our group as described previously [12, 13]. E6F6 exhibited the most striking therapeutic effects in several HBV-persistent mice. E6F6 regimen efficiently prevented initial HBV infection and reduced viral dissemination through FcγR-dependent phagocytosis in human-liver-chimeric mice and HBV transgenic (HBV-Tg) mice. Furthermore, an E6F6 epitope-based particulate vaccine, CR-T3-SEQ13 (HBsAg aa 113-135), was designed and showed efficacy in suppressing HBsAg in mice and cynomolgus monkeys (*Macaca fascicularis*) [14]. Polyclonal antibodies (pAbs) isolated from four CR-T3-SEQ13 vaccinated monkeys showed potent inhibition of HBsAg and HBV DNA levels in HBV-Tg mice.

In this study, we isolated mAbs from CR-T3-SEQ13 vaccinated monkeys, compared their therapeutic effects with E6F6, identified their epitopes on HBsAg and determined the pharmacokinetics (PKs) in cynomolgus monkeys. The results contribute to a deeper understanding of the therapeutic efficiency of this novel epitope-based vaccine. Meanwhile, humanized antibody hu1-23 exhibited more remarkable and prolonged HBsAg-suppressing effects than huE6F6-1 in HBV-Tg mice at two different doses. Furthermore, this study provided two more HBsAg-targeting mAbs as candidates for antibody engineering to improve the druggability such as stability, solubility, viscosity, PKs and immunogenicity for the successful development of an antibody-based therapy for chronic HBV.

## MATERIALS AND METHODS

### Vaccinated cynomolgus monkeys and peripheral blood mononuclear cells preparation

Cynomolgus monkeys were immunized with the CR-T3-SEQ13 protein as described previously [14]. Blood from two selected monkeys was collected in EDTA tubes at week 13. Peripheral blood mononuclear cells (PBMCs) were isolated from the blood collected with Ficoll-Paque PREMIUM (GE Healthcare Life Sciences) by density gradient centrifugation. The PBMCs were then frozen in 90% heat-inactivated fetal bovine serum (FBS) supplemented with 10% dimethyl sulfoxide and stored in liquid nitrogen until thawing for use in assays.

### Memory B cell enrichment, sorting and culture

The EasySep Human Pan Memory B cell Enrichment Kit (STEMCELL Technologies Inc.), and a human IgM and IgD negative selection kit (Miltenyi Biotec) were used to sort cynomolgus monkey memory B cells. The cells were then seeded at a density of 1.4 cells per well in 96-well tissue culture plates containing 220 mL of 40-Gy irradiated 293T cells expressing human CD40L (20 000 cells per well) and RPMI-1640 medium supplemented with 20% FBS and a cocktail of human cytokines (50 ng/mL IL-21, 25 ng/mL IL-2). The memory B cells were cultured for 14 days, and 50 μL of supernatant was then analyzed by anti-human IgG and anti-HBsAg ELISAs. The HBsAg-positive candidates were lysed in 50 μL of RLT buffer (Qiagen) and stored at −80°C for immunoglobulin gene cloning.

### Recovery of antibody sequences and antibody production

Variable region genes from the positive B cells were recovered by reverse transcription PCR (RT-PCR) using primers specific for cynomolgus monkey heavy and light chain variable regions as described previously [15]. Two rounds of PCR were performed by incorporating overlapping sequences at the 3′ and 5′ ends, allowing infusion cloning of the variable regions into human heavy and light chain vectors to generate monkey-human chimeric antibodies. Paired heavy and light chain constructs were co-transfected into the antibody-producing cell line Freestyle™ HEK-293F (Life Technologies) using PEI (Sigma) and grown in Freestyle™ 293 Expression Medium (Life Technologies). All cells were maintained in a humidified atmosphere of 5% CO_2_ at 37°C. After seven days of expression, the supernatants were harvested, and antibodies were purified by affinity chromatography using protein A resin as reported previously [16].

### Antibody humanization and construction of humanized-mouse reverse chimeric antibodies (rc-mAbs)

The process for antibody humanization was based on a Complementarity-determining region (CDR)-grafting strategy [17, 18]. Briefly, CDRs in the heavy and light chains of a cynomolgus monkey antibody were defined by IMGT®, the international ImMunoGeneTics information system® http://www.imgt.org (IMGT) [19]. The parental cynomolgus monkey antibody sequence and the most closely related human germline sequence were then aligned. Human IgG signal peptides and a Kozak sequence were engineered at the 5′ ends of both the VK and VH sequences. The humanized VK and VH fragments were then cloned into human IgG1 CK and CH vectors separately. For the rc-mAb generation, humanized monkey Fv sequences were separately cloned into mouse IgG1 CH and CK vectors. The processes for expression, purification and quantification of the engineered antibodies were the same as those for the chimeric cynomolgus monkey antibodies.

### Affinity measurement by SPR

Surface plasmon resonance (SPR) experiments were performed with a Biacore 3000 instrument (GE Healthcare) at 10°C. Immobilization was achieved with 45 μg/mL HBsAg in sodium acetate buffer at pH 4.5 using a flow rate of 5 μL/min. The target immobilization level of antigen was ~ 2 000 RU, and the immobilized HBsAg level was 2 400 RU. For affinity measurement, a series of seven concentrations (1.56–100 nM, 2-fold dilutions) of each antibody was injected across the sensor surface at a flow rate of 50 μL/min. The association time was 90 sec, followed by a 600-sec observation of dissociation during buffer wash. A regeneration step with a 5-μL injection of NaOH solution (50 mM) was used to break the remaining binding at a flow rate of 50 μL/min for 60 sec. SPR binding data were first processed with BIAevaluation (version 4.0.1, GE Healthcare).

### HBsAg ELISA and chemiluminescent quantitation

Recombinant HBsAg protein and CR-T3-SEQ13 protein were purchased from Beijing Wantai Biological Pharmacy Enterprise. Co., Ltd (Beijing, China). Synthesized SEQ13 and SEQ13-mutant peptides were purchased from Sangon Biotech Co., Ltd (Shanghai, China). Anti-HBsAg, anti-CR-T3-SEQ13 and anti-SEQ13 polypeptides were detected using indirect ELISA kits and chemiluminescent quantitation kits developed by Beijing Wantai Biological Pharmacy Enterprise Co., Ltd as described previously [14].

### Immunohistochemistry assay and intrahepatic HBsAg/HBcAg detection

Mouse livers were fixed in formalin (3.7% formaldehyde in PBS) for analysis. Intrahepatic HBsAg and hepatitis B core antigen (HBcAg) were detected using immunohistochemical staining with the anti-HBsAg mAb 83H12 and anti-HBcAg mAb 14C7-2, respectively. Both mAb 83H12 and 14C7-2 are homemade mouse monoclonal antibodies. The MaxVisionTM HRP-Polymer IHC Kit (Maixin, Fuzhou, China) was used. Homogenized sections were utilized for HBsAg and HBcAg detection, and the HBsAg and HBcAg levels in the homogenized liver tissues were detected with commercially available CLEIA and ELISA kits (Wantai, Beijing, China).

### *In vivo* efficacy in HBV-Tg mice

HBV-transgenic mice (pAAV/HBV1.2, genotype Ae, aged 8–10 weeks) were provided by Pei-Jer Chen (NTU, Taiwan) [20]. For *in vivo* treatment of HBV-Tg mice with mAbs, a specific dose of antibodies suspended in 200 μL of PBS was injected into mice (tail vein). The mice were bled via retro-orbital puncture at baseline and at the additional time points of 120 min and 1, 3, 5, 7, 14, 16, 20, 24, 27 and 31 days after mAb infusion. Mouse serum specimens taken at these time points were kept at −80°C until use. All mice were maintained under specific pathogen-free conditions in the Laboratory Animal Centre of Xiamen University, and experiments were conducted in accordance with the ‘Guide for the Care and Use of Laboratory Animals.’

### PK study in cynomolgus monkeys

Six cynomolgus monkeys were used to study antibody PKs. All the *in vivo* studies performed with cynomolgus monkeys were carried out by WuXi AppTec Co., Ltd (Suzhou, China). All cynomolgus monkey experiments in this study were reviewed and approved by the Institutional Animal Care and Use Committee of Xiamen University and WuXi AppTec’s Institutional Animal Care and Use Committee before initiating any activities involving animals. Three cynomolgus monkeys were assigned to the antibody group and given a single intravenous (i.v.) injection of 20 mg/kg hu1-23 or hu3-23. Blood samples were drawn prior to dosing on day 0 and at 0.083, 0.5, 1, 2, 4, 10, 24, 48, 72, 96, 144, 192, 240, 336, 408, 504 and 672 h after dosing. Data were collected and analyzed using the noncompartmental analysis model 200-202 of Phoenix WinNonlin version 6.3, as described previously [13].

**Figure 1:**
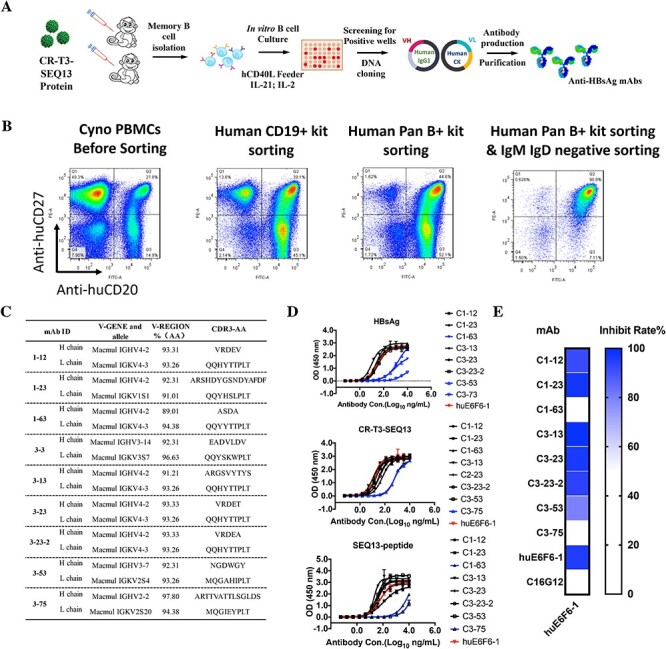
Characterization of HBsAg-SEQ13 specific antibodies from two vaccinated cynomolgus monkeys. (A) The strategy used to generate HBsAg-specific monkey mAbs from vaccinated cynomolgus monkeys. (B) Isolation of CD20^+^CD27^+^ memory B cells from cynomolgus monkeys. (C) Analyses of the sequences of nine anti-HBsAg antibodies. The antibodies named with ‘1-’ were from monkey #1, and those named with ‘3-’ were from the other monkey #3. (D) Evaluation of the binding activity of anti-HBsAg antibodies against HBsAg, CR-T3-SEQ13 and SEQ13-polypeptide. (E) Blocking effects of anti-HBsAg mAbs on the binding of huE6F6-1 with HBsAg. The data were expressed as the mean ± SD.

## RESULTS

### Isolation of anti-HBsAg antibodies from CR-T3-SEQ13-vaccinated cynomolgus monkeys

Two CR-T3-SEQ13-vaccinated cynomolgus monkeys were selected for isolation of mAbs from single memory B cells [14]. A short-term single memory B cell expansion system for cynomolgus monkeys was established to generate anti-HBsAg mAbs (Fig. 1A). Antibodies for analysis of the cell-surface markers CD20 and CD27 and surface IgG (sIgG) on monkey cells by Fluorescence-activated Cell Sorting (FACS) were selected as described previously [3]. To isolate sIgG-positive memory B cells from vaccinated monkeys, we carried out a preliminary sorting experiment using PBMCs from a healthy monkey. The EasySep™ Human Pan-B Cell Enrichment Kit was shown to effectively isolate CD20^+^ monkey B cells (gating 96.7%). After further negative selection with human IgM and IgD microbeads (Miltenyi Biotec.), the purity of isolated CD20^+^CD27^+^ B cells was 90.8, and 41.1% of these cells were gated as sIgG-positive cells (Fig. 1B). Single cells were sorted into 96-well plates containing HEK293-hCD40L feeder cells in a medium supplemented with 20% FBS. The cells were cultured in the presence of human IL-21 and IL-2 as described previously [21]. The two vaccinated monkeys were bled 21 days after a third booster immunization in week 13. The monkeys exhibited high anti-HBsAg titers of 3.68 × 10^3^ mIU/mL and 3.035 × 10^3^ mIU/mL at the bleeding time point (Fig. S1). Two vials containing 10 million PBMCs collected from each monkey were thawed and combined and memory B cells were sorted at a density of one cell per well and cultured in 96-well plates for two weeks, with 50 plates seeded per monkey. Nine anti-SEQ13 mAbs were generated all together. Among them, six antibodies utilized the IgH4-2 gene for heavy chain, and five of them used the IgK4-3 for light chain (Fig. 1C). The sequences of the nine antibodies were then converted into monkey-human chimeric antibodies (hu-IgG1), transiently expressed in HEK293 cells and purified (Fig. S2). Each of the recombinant antibodies demonstrated binding to HBsAg, CR-T3-SEQ13 and SEQ13-peptide, as measured by ELISA (Fig. 1D). The antibodies C1-63, C3-35 and C3-73 (blue lines) showed higher half-maximal effective concentrations (EC50s) for HBsAg, CR-T3-SEQ13, or SEQ13-peptide, while the others showed the same binding activity as huE6F6-1 (Fig. 1D). Furthermore, antibodies C1-12, C1-23, C3-13, C3-23 and C3-23-2, but not others, could completely block huE6F6-1 binding to HBsAg, suggesting these mAbs share the similar HBsAg binding sites with huE6F6-1 (Fig. 1E).

### Humanization of C1-23 and C3-23 and their viral suppression efficacy in HBV-Tg mice

**Figure 2:**
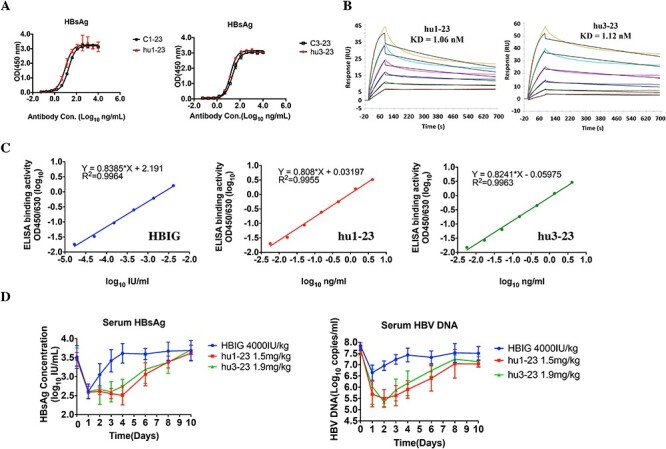
Humanization of C1-23 and C3-23 and the comparation of their viral suppression effects to HBIG in HBV-Tg mice. (A) Humanized 1-23 and 3-23 mAbs maintained binding activity similar to that of the parental chimeric antibodies in ELISA-based assays. (B) Representative SPR sensorgrams for the binding of hu1-23 and hu3-23 to HBsAg. (C) Dose–response analyses of the binding activity of hu1-23, hu3-23, and HBIG. Based on the correlation analyses, the binding activity of 4 000 IU/kg HBIG was equal to 1.5 mg/kg hu1-23 or 1.9 mg/kg hu3-23. (D) The comparison of therapeutic efficacy among equivalent doses of hu1-23, hu3-23 and HBIG in HBV-Tg mice (N = 5). The values represent the mean ± standard deviation from at least three experiments.

To select lead antibodies from each monkey for further characterization, we evaluated the antibody-mediated HBsAg and HBV DNA clearance efficacy in HBV-Tg mice (N = 4) for antibodies C1-12, C1-23, C3-13, C3-23 and C3-23-2. Antibodies C1-23 and C3-23 from each monkey showed the best clearance efficacy (Fig. S3). We then humanized C1-23 and C3-23 by CDR grafting [17, 18]. The humanized antibodies hu1-23 and hu3-23 maintained the comparable HBsAg binding activity as the parental antibodies C1-23 and C3-23, as measured by ELISA (Fig. 2A). The binding KD values to the HBsAg as measured via SPR (Biacore) for hu1-23 and hu3-23 was 1.06 nM and 1.12 nM, respectively (Fig. 2B). We then compared the therapeutic efficacy between hu1-23, hu3-23 and the commercial HBIG in an *in vivo* model. Dose–response analyses indicated that the HBsAg binding activity of 1 IU/mL HBIG was equal to 0.374 μg/mL hu1-23 and 0.484 μg/mL hu3-23 (Fig. 2C and S4). The *in vivo* efficacy study showed that hu1-23 or hu3-23 infusion induced a significant and prolonged suppression of serum HBsAg and HBV DNA than that of HBIG administrated at an equivalent dose (Fig. 2D).

### *In vivo* viral suppression mediated by antibodies 1-23 and 3-23 in HBV-Tg mice

**Figure 3:**
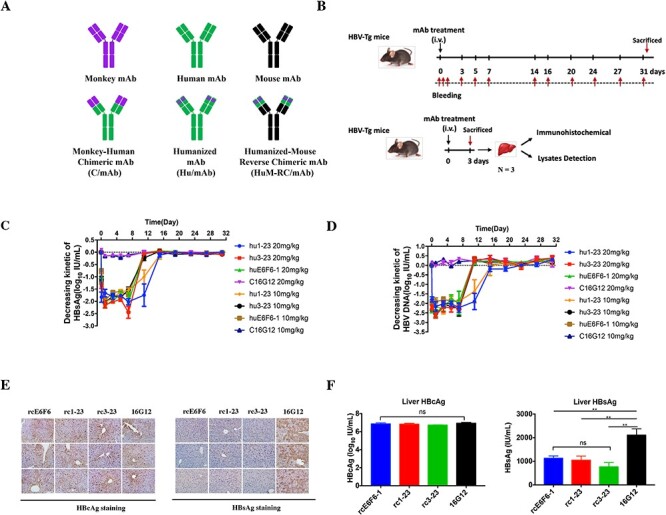
Therapeutic effects of 1-23 and 3-23 suppress HBV in HBV-Tg mice. (A) Different types of engineered mAbs used in this study: monkey–human chimeric mAb, cmAb, humanized mAb and humAb. Humanized-mouse reverses chimeric mAb, which contained the mouse IgG2a constant region, rc-mAb. (B) Study design for this figure. (C) and (D) Serum HBsAg and HBV DNA profiles of HBV-Tg mice (N = 5) after single infusions with different concentrations of hu1-23 or hu3-23. C16G12 was a mouse-human chimeric isotype control. Antibodies were used at a dose of 10 mg/kg or 20 mg/kg. The data were expressed as the mean ± SD Immunohistochemical staining for HBsAg and HBcAg in the liver of HBV-Tg mice (N = 3) after mAb infusion. Assays were performed 3 days after rc-mAb infusion. (E) Liver HBsAg and HBcAg profiles of HBV-Tg mice (N = 3) on day 3 after rc-mAb treatment. The data were expressed as the mean ± SD; ^*^^*^p < 0.01 compared to control; ns, not significant. (F) Serum HBsAg, HBV DNA profiles of HBV-Tg mice (N = 5) after different single rc1-23 and rc3-23 infusion. 16G12 was a mouse isotype control. Antibodies were used at dosage of 10 mg/kg g. The data were expressed as the mean ± SD.

At two dose levels of 10 and 20 mg/kg, a single infusion of hu1-23 or hu3-23 significantly decreased HBV DNA and HBsAg levels with comparable efficacy as huE6F6-1 in mice (Fig. 3C and D). For antibody hu3-23, the therapeutics efficacy is comparable to huE6F6-1 at both doses. However, for antibody hu1-23, single-dose treatment with hu1-23 at both doses (Day 10, 240 h post-infusion, orange line and blue line) cleared HBsAg and HBV DNA significantly more than treatment with huE6F6-1 and hu3-23, p < 0.01. Furthermore, after hu1-23 therapy at the dose of 20 mg/kg, the average decreasing kinetic of HBsAg and HBV DNA were at a continued 100 IU/mL rate for 10 days, 2 days longer than E6F6 and hu3-23. These results demonstrated that the antibodies 1-23 and 3-23 generated from CR-T3-SEQ13-vaccinated cynomolgus monkeys have the same or even more significant potential as huE6F6-1 for drug development.

To exclude the effect of human Fc-mediated effector function in the animal efficacy model, humanized-mouse reverse chimeric antibodies (rc-mAbs) consisting of the variable region of huE6F6-1, hu1-23 or hu3-23 and the murine Fc constant region were constructed (Fig. 3A). A homemade mouse monoclonal antibody 16G12 and its mouse-human chimeric version were used as the isotype control antibodies. Study design of this experiment was detailed in Fig. 3B. The rc-mAbs mediated anti-HBV efficacy was examined on intrahepatic HBV markers on liver tissues 3 days post-infusion via immunohistochemistry (N = 3) and liver lysates via CLEIA (N = 5). As shown in Fig. 3E and F, antibodies rc1-23 or rc3-23 did not change the intrahepatic levels of HBcAg; however, the results from both immunohistochemistry assays and lysate detection assays indicated that rc1-23 and rc3-23-treated mice exhibited significantly lower levels of intrahepatic HBsAg, which were similar to those observed in rcE6F6-1-treated mice. The results of a treatment experiment showed that rc1-23 or rc3-23 infusion induced significant and prolonged suppression of serum HBsAg and HBV DNA at the similar efficacy as in rcE6F6-1-treated mice (Fig. S5). These data suggest that both humanized and reverse chimeric versions of 1-23 and 3-23 exhibited comparable or even better therapeutic efficacy to those of E6F6-1.

### Anti-SEQ13 binding epitope and molecular docking of the antibody–antigen interactions

**Figure 4:**
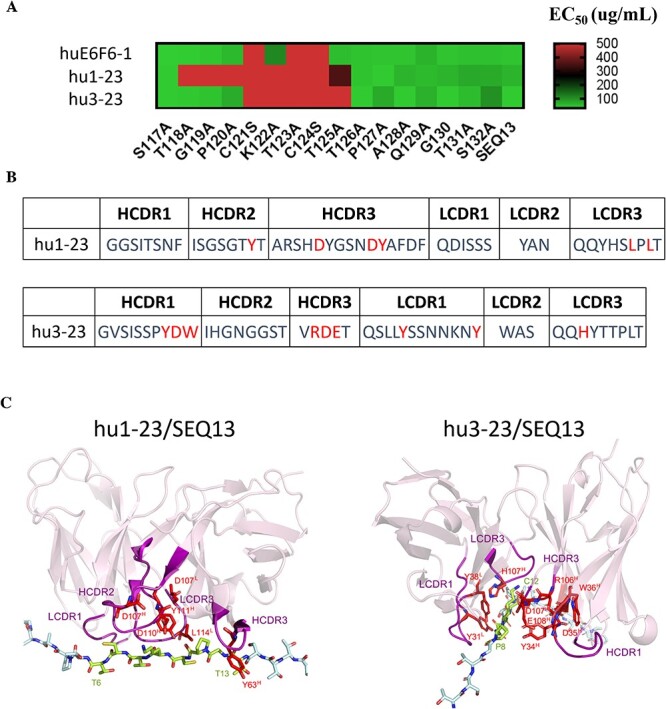
Detailed binding of hu1-23 and hu3-23 to HBsAg-SEQ13. (A) Evaluation of the EC50s of the antibodies for SEQ13 with single-site alanine-scanning mutagenesis. The cysteines at positions 121 and 124 were mutated to serine. (B) Critical amino acids contribute to the antibodies interacting with SEQ13. (C) Molecular docking of the interaction of hu1-23 and 3-23 against SEQ13.

The minimal binding sequence of the mouse antibody E6F6 is CK(R)TC, which represents amino acids (aa) 121-124 of HBsAg as described previously [12]. To map the binding epitopes of hu1-23 and hu3-23 on HBsAg, we measured the binding EC50s of huE6F6-1, hu1-23 and hu3-23 on different mutated-SEQ13. As shown in Fig. 4A, the binding epitopes of hu1-23 and hu3-23 were found to include the CK(R)TC sequence. The binding EC50s of huE6F6-1 to SEQ13-C121S, SEQ13-T123A or SEQ13-C124S were approximately 500 μg/mL or greater, and the binding epitope of hu3-23 largely overlaps with that of huE6F6-1 (Fig. 4A). However, hu1-23 recognized a longer epitope, which included 118T, G119, P120, K122 and T125, for hu3-23, K122 and T125 (Fig. 4A).

**Figure 5:**
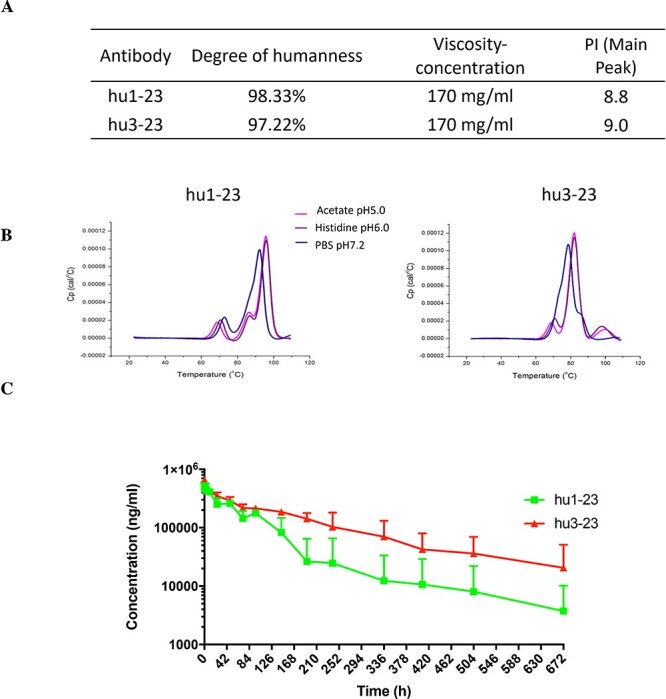
Physical property and *in vivo* PK profiles of hu1-23 and hu3-23. (A) Degree of humanness, viscosity-concentration and pI profile of hu1-23 and hu3-23. (B) DSC scan of mAbs in 25 mM Histidine (PH 6.0), 5% sucrose and 0.02% PS80. Scan rate: 200°C/h. DSC was performed with Microcal VP-DSC(GE). (C) Serum antibody concentrations (μg/mL) of hu1-23 and hu3-23 following a single i.v. infusion of 20 mg/kg mAb into cynomolgus monkeys (*n* = 3 animals per antibody). The data were expressed as the mean ± SD.

To identify the critical residues for antigen–antibody interaction, alanine-scanning mutagenesis was carried out in CDR regions of heavy and light chains. The effect of mutations on antigen binding was assessed by analyzing the ratio of EC50 values [22] (or relative EC50, rEC50) of purified antibodies by indirect ELISA against HBsAg. An amino acid is considered a critical residue if the rEC50 is less than 0.2. The essential residues identified in the experiments for hu1-23 and hu3-23 are shown in red: the six critical residues for hu1-23 are HCDR2 -Y63, HCDR3-D107/D112/Y113 and LCDR3-L114/L116, and the nine critical residues for hu3-23 are HCDR1-Y34/D35/W36, HCDR3-R106/D107/E108, LCDR1-Y31/Y38 and LCDR3-H111 (Fig. 4B). The binding surface of SEQ13 by hu1-23 and hu3-23 were generated by molecular docking using Discovery Studio (DS) (Supplementary Methods 2 and 3). First, structure models of hu1-23 and hu3-23 were generated by DS. We then identified the most likely binding models based on the modeling structure and the critical residues in CDRs of the humanized antibodies, and the key amino acids in SEQ13. As shown in Fig. 4C, HCDR3-TYR113 and ASP112 of hu1-23 suggested to interact with LYS122 of SEQ13 via a hydrogen bond and a salt bridge; and HCDR3-ASP112 of hu3-23 interacts with LYS122 of SEQ13 via a hydrogen bond and PI interaction. Moreover, LCDR3-TYR37 of hu3-23 may interact with THR123 from SEQ13 via a hydrogen bond (Fig. 4C).

### Physical property and *in vivo* PK of hu1-23 and hu3-23

Physical property study of hu1-23 and hu3-23 was conducted at WuXiAppTec., Ltd (WuXiAppTec, Wuxi, Jiangsu). Degree of humanness (hu1-23, 98.33%; hu3-23, 97.22%), viscosity concentration (hu1-23, 170 mg/mL; hu3-23, 170 mg/mL), thermostability and isoelectric points (pI; hu1-23, pI = 8.8; hu3-23, pI = 9.0) were summarized in Fig. 5A. Hu1-23 and hu3-23 were then buffer-exchanged into different buffers (acetate pH 5.0, histidine pH 6.0 and PBS pH 7.2) and subjected to differential scanning calorimetry (DSC) assay at T0 (the initial time) (Fig. 5B, Tables S1 and S2). In general, these studies revealed that hu1-23 and hu3-23 are stable enough for further development as a drug candidate.

The PK properties of hu1-23 and hu3-23 were examined in cynomolgus monkeys following a single i.v. administration of antibody (*n* = 3 animals per group). Blood samples were collected for up to 672 h (28 days) post-infusion. The serum concentration of anti-HBsAg antibodies was measured. The average serum concentration-time profiles and relevant PK parameters are shown (Fig. 5C and Table 1). For hu1-23, a serum antibody concentration of more than 100 μg/mL was measured for approximately 400 h (t_1/2_ = 81.3 ± 78.7 h), and this serum concentration threshold was exceeded for more than 672 h for hu3-23 (t_1/2_ = 134.0 ± 94.6 h). Taken together, these results indicate that hu1-23 and hu3-23 possess the desirable physical properties and pharmacological characteristics as potential drug candidates.

**Table 1:** Pharmacokinetic parameters of hu1-23 and hu3-23 in cynomolgus monkeys

Test article	hu1-23	hu3-23
Dose(mg/kg)	20.0	20.0
C_0_ (ng/mL)	491 350 ± 119 848	659 429 ± 89 752
T_1/2_ (h)	81.3 ± 78.7	134.0 ± 94.6
Vd_ss_ (L/kg)	0.0487 ± 0.00894	0.0497 ± 0.0143
Cl (mL/min/kg)	0.00971 ± 0.00303	0.00490 ± 0.00255
T_last_ (h)	672 ± 0	672 ± 0
AUC_0-last_ (ng.h/mL)	36 480 687 ± 12 092 238	72 786 127 ± 26 164 236
AUC_0-inf_ (ng.h/mL)	36 769 777 ± 11 998 175	79 419 210 ± 33 500 012
MRT_0-last_ (h)	87.4 ± 50.3	161 ± 68.8
MRT_0-inf_ (h)	93.2 ± 47.2	210 ± 137
AUC_Extra_ (%)	0.840 ± 1.45	6.21 ± 9.74
AUMC_Extra_ (%)	7.74 ± 13.3	18.3 ± 27.0

PK parameters of hu1-23 and hu3-23 in cynomolgus monkeys, calculated using the noncompartmental analysis model 200-202 of Phoenix WinNonlin version 6.3.

## DISCUSSION

It is challenging to overcome immune tolerance in patients with HBV and produce an anti-HBsAg antibody response that can mediate HBsAg clearance [2]. Therefore, develop novel treatment strategies is necessary to overcome HBsAg-related immune tolerance in CHB patients. E6F6, a mouse antibody recognizing the evolutionarily conserved sA epitope (119-125, GPCK(R)TCT) on HBsAg, displays more prolonged HBV suppression effects than mAbs binding to other epitopes we tested in HBV-Tg mice. Single-dose administration of E6F6 can profoundly suppress HBsAg and HBV DNA levels for several weeks. E6F6-based immunotherapy can facilitate anti-HBV T-cell response in hydrodynamic injection-based HBV carrier mice as described in our previous study [12]. The CR-T3-SEQ13 protein, an E6F6 epitope-based particulate vaccine, effectively suppressed and cleared HBsAg in mice and cynomolgus monkeys after vaccination. Polyclonal antibodies induced in vaccinated cynomolgus monkeys were shown to more effectively suppress and clear HBsAg in HBV-Tg mice than HBIG but not as potent as E6F6 [14]. In addition to the polyclonal antibodies response, evaluation of the *in vivo* HBV therapeutic efficacy of the monoclonal antibodies generated from the vaccinated subjects is necessary to dissect the detailed immune response to the therapeutic vaccine.

In this study, two lead mAbs from two CR-T3-SEQ13 vaccinated cynomolgus monkeys, 1-23 and 3-23, were selected after multiparameter characterization. The therapeutic efficacy of the humanized mAbs, hu1-23 and hu3-23, was better than an equivalent dose of hepatitis B immune globulin (HBIG) in HBV-Tg mice. Comparable anti-HBV efficacy was observed for hu1-23, hu3-23, huE6F6-1 and their corresponding reverse chimeric versions (rc-mAbs). Our result suggests that vaccination of cynomolgus monkeys with CR-T3-SEQ13 can induce E6F6-like mAbs that efficiently mediate HBsAg clearance in HBV-Tg mice. HBsAg levels were suppressed by these E6F6-like mAbs. However, hu1-23 exhibited more remarkable and prolonged HBsAg-suppressing effects than huE6F6-1 and hu3-23 in HBV-Tg mice at dose levels of 10 and 20 mg/kg. Although the half-life of the antibody in mouse and cynomolgus monkeys may differ, these results still suggest that hu1-23 may clear the HBsAg much more efficacy. PK studies showed that hu1-23 has a shorter half-life in cynomolgus monkeys than that of hu3-23 and huE6F6-1 [13]. It would be interesting to engineer hu1-23 with an enhanced half-life, pH-dependent-HBsAg binding and improve therapeutic potency. Epitope mapping studies suggest that the epitopes recognized by the two cynomolgus monkey mAbs (hu1-23 and hu3-23) and huE6F6-1 were overlapping, but amino acids flanking the huE6F6-1 epitope CK(R)TC may also contribute to the interactions between hu1-23 or hu3-23 and HBsAg, suggesting the epitopes of the two antibodies and huE6F6 are identical.

It is interesting that several monoclonal monkey antibodies have IGHV4-2 and IGKV4-3. Although they have the same variable genes, the sequences of the CDRs are pretty different (Fig. 1C). Besides the similar v-genes, six of these nine antibodies have a short HCDR3 (3-7 aa). It is hard to suggest antibody convergence, as we do not have enough antibodies. However, it is still interesting why these antibodies generated from different subjects choose similar variable genes and short HCDR3. We will screen more antibodies from more vaccinated subjects and try to figure out what happened. There is also a lack of immunological studies to figure out the immune effect after treatment of these E6F6-like antibodies, and we hope to have the opportunity to verify this in the future.

Nonhuman primates including cynomolgus monkeys are good source of therapeutics mAbs with a higher degree of humanness than mouse antibodies. The humanization of monkey monoclonal antibodies is much easier than mouse antibodies. Researchers have developed several strategies to generate functional mAbs from vaccinated nonhuman primates which include antibody phage display, single B cell cloning and next-generation sequencing [15, 23, 24]. Culture conditions stimulating memory B cells *in vitro* have also been widely used to generate functional antibodies from vaccinated or virus-infected subjects, such as those infected with HIV, severe acute respiratory syndrome coronavirus (SARS-CoV), SARS-CoV-2, dengue virus, hepatitis E virus or HBV [25, 26]. In this study, monkey monoclonal antibodies were generated from the vaccinated cynomolgus monkeys via stimulation of memory B cells *in vitro*. To our knowledge, there are few reports on stimulating nonhuman primate memory B cells in culture [27–29] and fewer reports on generating nonhuman primate mAbs from a short-term B cell culture protocol [30]. Our data indicated that cynomolgus monkey memory B cells could be effectively stimulated by human IL-21/IL-2 and human CD40L^+^ feeder cells *in vitro*. The high homology between antibodies from nonhuman primates and human antibodies contributes to the humanization of the monkey mAbs.

Although our previous studies have confirmed that E6F6 and its humanized antibodies are effective in HBV suppression [13], more candidate antibody molecules with comparable or even better functions are needed as backups [31–33]. Therefore, hu1-23 and hu3-23 are novel antibodies that may serve as anti-HBsAg therapeutic candidates for CHB patients. Moreover, the generation of antibodies from the epitope-based vaccinated subjects may be an alternative approach for novel antibody drug discovery.

## Data Availability Statement

Data is contained within the article can be available online.

## Conflict of Interest Statement

None declared.

## Funding

This study received the following funding: the National Natural Science Foundation of China (31870925), the National Science and Technology Major Project (2017ZX10202203-009-003) and the Fujian Provincial Medical Innovation Foundation (2017-CXB-20).

## Supplementary Material

Supplementary-final_ABT_tbab020
