# Supplementary material for: Novel monkey mAbs induced by a therapeutic vaccine targeting the hepatitis B surface antigen effectively suppress hepatitis B virus in mice
Source: Antib Ther. 2021 Sep 29;4(4):197–207. doi: 10.1093/abt/tbab020 (PMC8499627; doi:10.1093/abt/tbab020)
Supplement: Supplementary-final_ABT_tbab020 [file supplementary-final_abt_tbab020.docx]

**Supplementary Materials**

**for**

**Novel monkey mAbs induced by a therapeutic vaccine targeting the hepatitis B surface antigen effectively suppress hepatitis B virus in mice**

Yuanzhi Chen ^1, 2^, Xinchu Xiang ^1, 2^, Ruoyao Qi ^1, 2^, Yiwen Wang ^1, 2^, Yang Huang ^1, 2^, Min You ^1, 2^, Yangfei Xian ^1, 2^, Yangtao Wu ^1, 2^, Rao Fu ^1, 2^, Ciming Kang ^1, 2^, Jixian Tang ^1, 2^, Hai Yu^1, 2^, Tianying Zhang ^1, 2^, Quan Yuan^1, 2,^ ^*^ Wenxin Luo ^1, 2, *^, Ningshao Xia^1, 2^

^1^ State Key Laboratory of Molecular Vaccinology and Molecular Diagnostics, School of Public Health & School of Life Science, Xiamen University, Xiamen, China.

^2^ National Institute of Diagnostics and Vaccine Development in Infectious Diseases, School of Public Health & School of Life Science, Xiamen University, Xiamen, China.

**Supplementary Figures/Tables**

**Fig. S1.** Anti-HBsAg titers of two selected cynomolgus monkeys determined as described. One monkey was labeled as #1, and the other monkey was labeled as #3. Twenty milliliters of blood from each monkey was collected on week 13.

**Fig. S2.** Reducing PAGE gel and nonreducing PAGE gel showing monkey anti-HBsAg mAbs

**Fig. S3.** Evaluation of antibody-mediated HBsAg and HBV DNA clearance effects in HBV-Tg mice (N=4) mediated by the selected monkey-human chimeric antibodies.

1. HBsAg level. (B) HBV DNA level. The data are expressed as the mean ± SD.

**Fig. S4.** Dose-response analyses of the binding activity of hu1-23, hu3-23 and HBIG. The data are expressed as the mean ± SD.

**Fig. S5.** Serum HBsAg, HBV DNA profiles of HBV-Tg mice (N=5) after different single rc1-23 and rc3-23 infusion. 16G12 was an mouse isotype control. Antibodies were used at dosage of 10 mg/kg g. The data were expressed as the mean±SD.

**Table S1. Tm value of hu1-23**

| Buffer | hu1-23 | | |
| --- | --- | --- | --- |
|  | Tm onset (℃) | Tm1 (℃) | Tm2 (℃) |
| Acetate pH5.0 | 61.7 | 68.9 | 95.6 |
| Histidine pH6.0 | 63.8 | 71.0 | 96.1 |
| PBS 7.2 | 65.6 | 72.9 | 92.4 |

**Table S2. Tm value of hu3-23**

| Buffer | hu3-23 | | |
| --- | --- | --- | --- |
|  | Tm onset (℃) | Tm1 (℃) | Tm2 (℃) |
| Acetate pH5.0 | 61.6 | 68.8 | 82.2 |
| Histidine pH6.0 | 62.8 | 71.1 | 82.3 |
| PBS 7.2 | 65.7 | 78.5 | No data |

**Supplementary methods**

1. **Statistical analysis**

Statistical analyses were performed with Prism 7.0 (GraphPad software). Differences were determined to be significant at p < 0.05 using a two-tailed Student’s test and a two-tailed Mann-Whitney log-rank test. In all figures, all of the data with error bar are expressed as the mean ± SD.* indicates p < 0.05; and ** indicates p < 0.01; ns, not significant.

1. **Antibody structure modeling**

Antibody structure modeling of hu1-23 and hu3-23 Fv were generated using Discovery Studio® Software 2017 R2(DS). Homology template structures of the top hits predicted by the Identify Framework Templates protocol were selected. These structures were used as input structures, becoming the foundation on which the Model Antibody Framework protocol built a human antibody Fab structure. The Model Antibody Loops protocol identifies templates and manufactures models for CDRs using Hidden Markov Models(HMM). We used this protocol to rebuild the CDR loops. Finally, the homology model of hu1-23 and hu3-23 Fv were subject to energy minimization using the CHARMM force field. In this way, we developed a lowest energy structure for docking and further analysis.

1. **Antibody Docking**

Rigid body docking of hu1-23, hu3-23 and SEQ13 was performed using the ZDock algorithm as described(1). Next, hierarchical sets of clusters with different docked poses were generated according to antibody position. Rescoring the poses was done using the ZRank scoring function based on the electrostatics, van der Waals, and desolvation energy terms.

Docked poses were filtered based on the interacting sites of antibodies and SEQ13 we identified. Next, all ranked poses with the ZRank scores lower than -55 were selected from the most significant ten clusters. These were input into the RDock procedure. Input docked structures were refined by eliminating minor clashes and optimizing polar and charge interactions, then re-ranked according to the electrostatic and solvation energy terms(2). All docked poses were performed by RDock with default parameters.

**References**

1. Chen, R. and Weng, Z. (2002) Docking unbound proteins using shape complementarity, desolvation, and electrostatics. *Proteins*, **47**, 281-294.

2. Chen, R., Li, L. and Weng, Z. (2003) ZDOCK: an initial-stage protein-docking algorithm. *Proteins*, **52**, 80-87.
